# Workers' Decisions to Disclose a Mental Health Issue to Managers and the Consequences

**DOI:** 10.3389/fpsyt.2021.631032

**Published:** 2021-03-26

**Authors:** Carolyn S. Dewa, Jaap van Weeghel, Margot C. W. Joosen, Petra C. Gronholm, Evelien P. M. Brouwers

**Affiliations:** ^1^Department of Psychiatry and Behavioral Sciences, University of California, Davis, Davis, CA, United States; ^2^Tranzo, Scientific Center for Care and Wellbeing, Tilburg University, Tilburg, Netherlands; ^3^Health Services and Population Research Department, King's College London, Institute of Psychiatry, Psychology and Neuroscience, London, United Kingdom

**Keywords:** stigma, mental health, managers, disclosure, workplaces, workers

## Abstract

**Background:** Stigma can be a barrier to accessing effective interventions and work accommodations for mental illnesses. Fear of stigma's concomitant prejudice and discrimination can inhibit workers from asking for help. Thus, it may be important to develop effective interventions addressing workplace stigma. To identify important targets for these interventions, this study addresses three questions: (1) what proportion of workers experiencing mental health issues disclosed their mental health issue to their managers, (2) what factors did they identify as contributing to their disclosure decisions, and (3) what were the consequences of their decisions?

**Methods:** The dataset is comprised of responses from respondents who were randomly drawn from a nationally representative sample of working Dutch adults who completed a web-based survey in February 2018. Respondents indicating they either had or have mental health issues were asked three sets of questions focusing on: (1) Did you disclose your mental health issue to you manager? (2) For what reasons did you disclose/not disclose the issue? (3) What were the consequences of your disclosure decision?

**Results:** About 73% of respondents with lived experience with mental health issues told their managers about their mental health issue. The structure of the survey questions identified four groups of workers who either: (1) *disclosed* and had a *positive* experience (64.2%), (2) *disclosed* and had a *negative* experience (9.0%), (3) *did not disclose* and had a *positive* experience (22.6%), or (4) *did not disclose* and had a *negative* experience (4.2%).

**Conclusion:** Our results reflect workers' diverse preferences for disclosing their mental health issues to their managers. Understanding both the factors that contributed to the decision to disclose and the consequences of disclosure decisions could help to better target workplace educational programs and interventions to address workplace stigma. Our findings suggest that addressing workplace stigma may not be as straightforward as requiring all employees to receive anti-stigma education. Rather, education should support workers to make the appropriate disclosure decision based on their workplace contexts. Future research is needed to understand the optimal ways for workers struggling with mental health issues to ask and receive help if they need it.

## Workers' Decisions to Disclose a Mental Health Issue to Managers and the Consequences

During a 30-day period, an estimated 11% of workers experience a mental disorder (1). Their disorders often hinder their work functioning (1). This can lead to significant work productivity losses (2). In addition, there is evidence that even when symptoms are in remission, work limitations may continue (3). This suggests that both during and after an episode of a mental disorder, workers may need help at work. This need for help may be underscored by the fact that those with a history of work disability are significantly more likely to have a future one (4). Thus, there is a rationale for work-based interventions for mental disorders.

During the past decade, there has been an increase in the development of work-based interventions to prevent and decrease mental disorder related work disability (5). These interventions include models to increase access to treatments and work accommodations (6, 7). There is evidence that work-based interventions can decrease the likelihood of sickness absences (8). Studies have reported that workers with mental disorders who receive treatment are more productive at work than workers who do not (9). Furthermore, workers who perceive that they have managers who are supportive and open to working with employees who have mental disorders are less likely to have work absences (10).

However, there is equivocal evidence that all workers who could benefit from treatment and/or supports access them (11). For example, Canadian studies found that almost half of workers who could benefit from mental health services did not access any (9, 11). A US study observed that compared to workers with physical disorders, those who experienced depression were less likely to report receiving work accommodations (12). A Dutch study found that a third of workers with mental disorders reported receiving work accommodations (8). At the same time, compared to workers with chronic physical disorders such as asthma/Chronic Obstructive Pulmonary Disease (COPD), diabetes, and musculoskeletal disorders, those Dutch workers with mental disorders were more likely to have work accommodations (8).

Stigma has been identified as a barrier to asking for help (13, 14). In the workplace, receipt of work accommodations relies on the worker's willingness to ask managers and supervisors for help; this requires disclosing a mental disorder (15, 16). Stigma related to mental illnesses can prevent workers from asking for help and disclosing that they are struggling with their mental health because they fear public stigma (i.e., mental illness related prejudice and discrimination from the environment) (15, 17). Disclosing a mental disorder potentially exposes a worker to stigma-related negative behaviors from managers and co-workers that could include social rejection, prejudice, discrimination, and harassment (14, 18, 19).

In addition to public stigma, workers with a mental disorder may also experience internalized or self-stigma (20). Self-stigma is a result of a person believing and applying the external prejudices associated with mental disorders to themselves (14, 21). Self-stigma leads people to devalue themselves (20). In turn, this can reduce their likelihood of seeking help (14).

Few workplace studies have focused on experiences of workplace stigma related to mental illness and disclosure (17). Rather, studies generally focus on attitudes about disclosure. Fewer studies have examined the actual consequences of decisions about disclosing a mental illness to managers. If stigma is a significant barrier for workers to accessing help at work, more information regarding disclosure is required to guide the development of ways to tackle this barrier. Stigma reduction is one of the strategies to support workers at work and to help workers obtain effective accommodations when they return to work from a work disability leave.

### Purpose

This study uses a subsample that was randomly drawn from a representative sample of adults in the Dutch labor force to examine the experiences of workers who had a mental health issue, their decisions to disclose their struggles to their managers, and the consequences of their decisions. Specifically, our study addresses the following sets of questions. Among those who have experienced a mental health issue, (1) what proportion disclosed their mental health issue to their managers, (2) what factors did they identify as contributing to their disclosure decisions, and (3) what were the consequences of their decisions? Our results can be useful in understanding what contributes to disclosure decisions and further our understanding about the consequences of disclosure choices.

## Methods

### Study Population

The data are from the February 2018 *Longitudinal Internet Studies for the Social Sciences* (LISS) panel that is administered by CentERdata. The panel was developed through a cooperation of CentERdata and Statistics Netherlands. The LISS panel sample consists of 5,000 households and 7,357 panel members. LISS panel members have given informed consent to participate in monthly questionnaires such as the one for this study. From the LISS panel, participants over the age of 18 years who identified their most important daily activity as paid employment were selected as potential recipients for this study. From this pool, a random sample of 1,671 participants was selected to receive the link to a web-based questionnaire. The response rate was 73.5% (*n* = 1,228). There were 1,224 respondents who voluntarily completed the survey, indicated they were in the labor force (i.e., were employed for pay or looking for employment) and were not in management positions. This study focuses on the disclosure experiences of the 332 workers who indicated they have experienced mental health issues either in the past or at the time of the survey. This group comprised 27% of the total study sample. The study dataset was de-identified by CentERdata prior to its use for the analysis. The University of California, Davis' Institutional Review Board reviewed the study protocol.

### Outcome Variables

#### Decision to Disclose Mental Health Problem (Q1)

**Figure 1:**
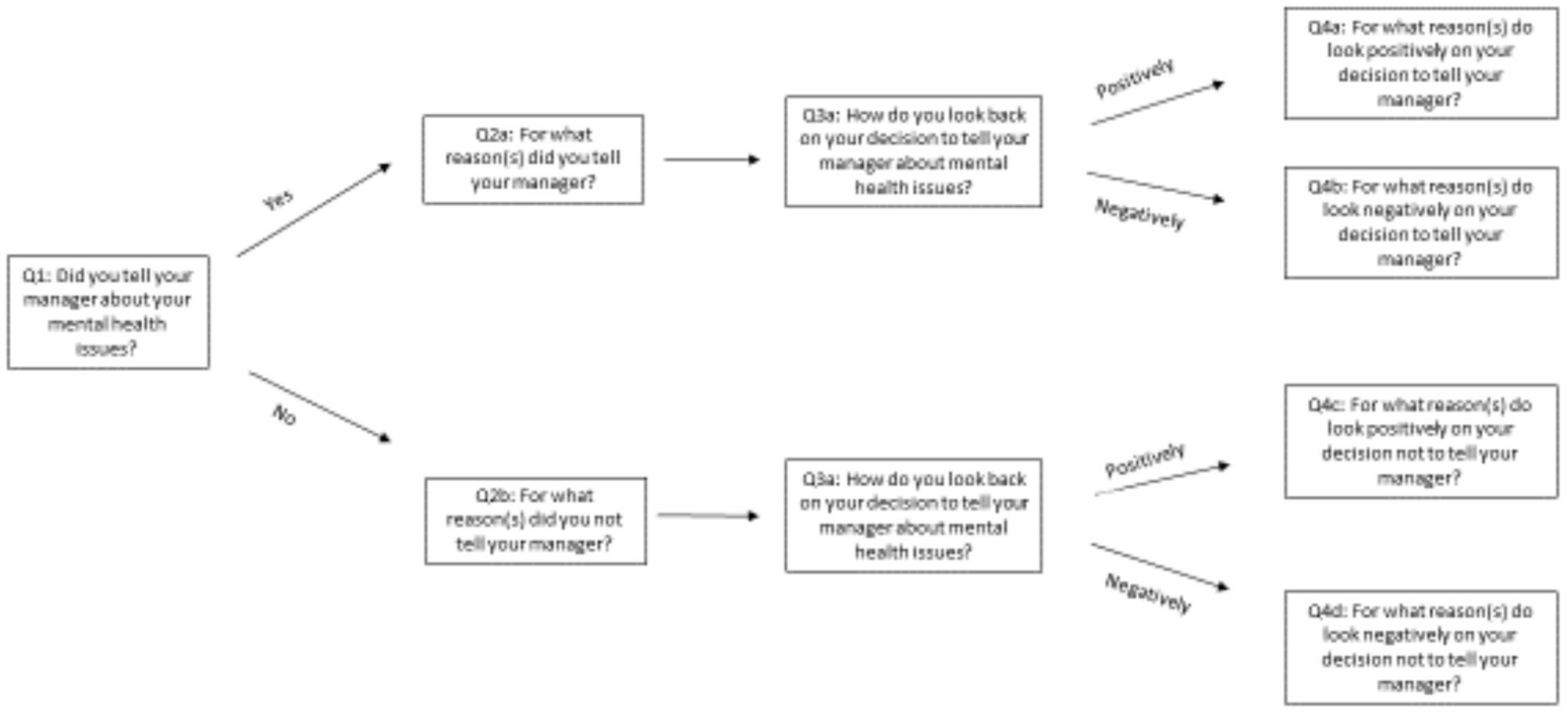
Survey questions analyzed.

The first question in the series of questions about workers' experiences with disclosing began with inquiring whether a mental health issue was disclosed to their managers (Figure 1).

#### Reasons for a Positive Disclosure Decision (Q2a)

Subsequent questions were based on responses to Q1. The response choices included in the questionnaire were adapted from studies conducted by Brohan et al. (17) and Dewa (22) that examined workers' attitudes toward disclosing a mental disorder to their managers (see **Tables 2**, **3** for list).

If there was an affirmative disclosure response to Q1, the respondent was asked the reason(s) for disclosing (Q2a). Respondents could identify as many reasons as applied. Applying concepts from educational theory, responses were aggregated into one of two categories: (1) intrinsic or (2) extrinsic (23). Educational theory suggests that different educational approaches are needed to address the two different types of motivating factors. Intrinsic factors are those that are motivated by the inherent satisfaction that they bring to the person doing them. For Q2a, intrinsic factors included disclosure reasons motivated by a sense of responsibility and positive feelings toward managers (listed in **Table 2**). In contrast, extrinsic factors represent activities done in response to either external encouragement or discouragement (23). Extrinsic factors are those that are motivated by anticipated reactions of others, incentives offered by the organization or manager, or advice from a trusted source. For Q2a, extrinsic factors included motivation based on the belief that the manager would notice the problem, the positive example others who disclosed and benefitted from disclosing, a recommendation from an occupational health physician, the desire to obtain either work accommodations, treatment during work hours, solutions offered through organizational policies or to prevent having to report sick (listed in **Table 2**).

#### Reasons for a Negative Disclosure Decision (Q2b)

If Q1 was answered in the negative (i.e., did not tell their managers), respondents were queried about the reasons for their negative response (Q2b). Respondents could choose as many reasons as applied.

Again applying concepts from educational theory, responses were aggregated into either: (1) intrinsic factors or (2) extrinsic factors (listed in **Table 2**) (23). For Q2b, intrinsic factors included the belief that it would not make a difference, a preference to deal with it alone, discomfort or embarrassment from disclosing, and failure to see a reason to disclose. For Q2b, extrinsic factors included fear of negative career effects, negative impacts on relationships, and potential loss of friends.

#### Actual Disclosure Decision Consequences (Q3a and Q4a–d)

Respondents were also asked about the consequences of disclosing—both positive and negative (Q3a). They were then asked for the factors that contributed to their positive or negative experiences (Q4a–d).

### Descriptive Variables

From responses to demographic questions, descriptive variables were created. They included sex (male/female), age ( ≤ 34, 35–44, 45–54, ≥55 years), marital status (single/never married, married/cohabiting, divorced/separated/widowed), and educational attainment (≤ high school yes/no).

### Analyses

χ^2^ tests were used to examine the differences in the characteristics of those who did and did not disclose their mental health issue to their manager. The 95% confidence intervals were calculated for the prevalence rates for the responses to the questions that asked about (1) the reasons for disclosing to a manager and (2) the consequences of disclosing (or not disclosing). Given small cell sizes, additional statistical analyses were not performed.

## Results

Table 1 indicates that there were significant differences between those who did and did not disclose with regard to age, marital status and educational attainment. Compared with those who disclosed, those who did not were significantly younger [$\chi_{\left(3\right)}^{2}$ = 8.0, *p* = 0.05], more likely to be single/never married [$\chi_{\left(2\right)}^{2}$ = 9.5, *p* = 0.02], and more likely to have more than a high school degree [$\chi_{\left(1\right)}^{2}$ = 4.2, *p* = 0.04].

**Table 1:** Demographic characteristics by disclosure to manager.

	**Total**		**Disclosed to manager**		**Did not disclose to manager**		**Test of significant differences between characteristics of workers who did and did not disclose**
	**%**	***n***	**%**	***n***	**%**	***n***	
**Total**	**100%**	**332**	**73.2%**	**243**	**26.8%**	**89**	
**Sex**							
Male	31.3%	104	30.5%	74	33.7%	30	$\chi_{\left(1\right)}^{2}$ = 0.32, *p* = 0.57
Female	68.7%	228	69.6%	169	66.3%	59	
**Age**							
* ≤* 34 years	24.7%	82	21.0%	51	34.8%	31	$\chi_{\left(3\right)}^{2}$ = 8.0, *p* = 0.046
35–44	26.5%	88	28.4%	69	21.4%	19	
45–54	26.2%	87	28.4%	69	20.2%	18	
≥55 years	22.6%	85	22.2%	54	23.6%	21	
**Marital Status**							
Married/co-habiting	55.4%	184	54.7%	133	57.3%	51	$\chi_{\left(2\right)}^{2}$ = 9.5, *p* = 0.0088
Separated/divorced/cohabiting	13.6%	45	16.9%	41	4.5%	4	
Single, never married	31.0%	103	28.4%	69	38.2%	34	
**Educational Attainment**							
High school degree or less	25.6%	85	22.6%	55	33.7%	30	$\chi_{\left(1\right)}^{2}$ = 4.19, *p* = 0.041
More than high school degree	74.4%	247	77.4%	188	66.3%	59	

### Disclosure

Table 2 contains the reasons for the disclosure decision. A significantly larger proportion of respondents disclosed their mental health issues than who did not [73.2% (*n* = 243), 95% CI: 68.1–77.9 vs. 26.8% (*n* = 89), 95% CI: 22.1–31.9]. The structure of the survey questions identifies four groups of workers who either: (1) *disclosed* and had a *positive* experience [64.2% (*n* = 213), 95% CI: 58.7–69.3], (2) *disclosed* and had a *negative* experience [9.0% (*n* = 30), 95% CI: 6.2–12.6], (3) *did not disclose* and had a *positive* experience [22.6% (*n* = 75), 95% CI: 18.2–27.5], or (4) *did not disclose* and had a *negative* experience [4.2% (*n* = 14), 95% CI: 2.3–7.0].

**Table 2:** Disclosure decision and reasons by experience with mental health problems.

	**Reasons for decision**		
	**%**	***n***	**95% CI**
**Disclosed to Manager**	**73.2%**	**243**	**68.1, 77.9**
Reasons for disclosing^*^			
**Intrinsic Factors**	**74.9%**	**182**	**69.0, 80.2**
Good relationship with manager	43.6%	106	37.3, 50.1
Feel responsible	46.9%	114	40.5, 53.4
Consistent with open personality	33.7%	82	27.8, 40.1
Did not want to hide	27.2%	66	21.7, 33.2
**Extrinsic Factors**	**76.1%**	**185**	**70.3, 81.3**
Seen how others benefited	3.3%	8	1.4, 6.4
Manager would be able to tell	19.8%	48	14.9, 25.3
Obtain work accommodations	21.4%	52	15.4, 27.1
Company doctor	7.8%	19	4.8, 11.9
Prevent having to report sickness absence	16.9%	41	12.4, 22.2
No choice, reported sick	39.9%	97	33.7, 46.4
Get time off for treatment during work	15.6%	38	11.3, 20.8
Organizational policies	–	–	–
**Did not disclose to manager**	**26.8%**	**89**	**22.1, 31.9**
Reasons for not Disclosing^*^			
**Intrinsic factors**	**69.7%**	**62**	**59.0, 80.0**
Would not affect work	27.0%	24	18.1, 37.4
Prefer to deal with it alone	44.9%	40	34.4, 55.9
Feel uncomfortable or embarrassed	13.5%	12	7.2, 22.4
Problems not serious	14.6%	13	8.0, 23.7
Did not realize needed help	13.5%	12	7.2, 22.4
Did not see a reason	16.9%	15	9.8, 26.3
**Extrinsic factors**	**34.8%**	**31**	**25.0, 45.7**
Fear of negative effect on career	16.9%	15	9.8, 26.3
Fear of affecting relationship with manager	7.9%	7	3.2, 15.5
Fear of losing friendships	–	–	–
Seen others have negative experience	7.9%	7	3.2, 15.5
Wouldn't want to be treated differently	–	–	–

* *Categories do not sum to 100% because respondents identified all factors that applied*.

### Reasons for Disclosing

When asked for the reasons for their decision, respondents who disclosed their mental health issues to their managers were as likely to point to intrinsic factors as they were to extrinsic factors [74.9% (*n* = 182), 95% CI: 69.0–80.2 vs. 76.1% (*n* = 185), 95% CI: 70.3–81.3]. Among the primary intrinsic motivating factors were a good relationship with their manager [43.6% (*n* = 106), 95% CI: 37.3–50.1] and feeling a responsibility to disclose [46.9% (*n* = 114), 95% CI: 40.5–53.4]. The primary extrinsic influencing factor was the fact that when they reported sick, they felt compelled to disclose [39.9% (*n* = 97), 95% CI: 33.7–46.4].

### Reasons for Not Disclosing

Among those who chose not to disclose, there were significantly more who were influenced by intrinsic factors than extrinsic factors [69.7% (*n* = 62), 95% CI: 59.0–80.0 vs. 34.8% (*n* = 31), 95% CI: 25.0–45.7]. Significant intrinsic factors included preferring to deal with the issues alone [44.9% (*n* = 40), 95% CI: 34.4–55.9] and believing their work would not be affected by their mental health issues [27.0% (*n* = 24), 95% CI: 18.1–37.4]. Fear for their careers [16.9% (*n* = 15), 95% CI: 9.8–26.3] was one of the primary extrinsic motivations for those who chose not to disclose.

### Consequences of Disclosing

Among workers who disclosed their mental health issues, 87.7% (*n* = 213) (95% CI: 82.8–91.5) indicated that their disclosure led to a positive experience (Table 3). The most frequently reported positive experience was managerial support [69.5% (*n* = 148), 95% CI: 62.8–75.6]. In contrast, 12.4% (*n* = 30) (95% CI: 8.5–17.2) indicated they had a negative experience from disclosing primarily as a result of not receiving managerial support [66.7% (*n* = 20), 95% CI: 47.2–82.7] or losing their job as a consequence [46.7% (*n* = 14), 95% CI: 36.1–57.5].

**Table 3:** Disclosure decision experiences.

	**%**	***n***	**95% CI**
Chose to disclose to manager^*^	**100%**	**243**	
**Positive experience from disclosing**	**87.7%**	**213**	**82.8, 91.5**
Supported by manager	69.5%	148	62.8, 75.6
Improved workplace relationships	22.5%	48	17.1, 28.7
Led to positive workplace changes	33.3%	71	27.0, 40.1
Did not need to hide	36.2%	77	29.7, 43.0
Could be a positive example	12.7%	27	8.5, 17.9
**Negative Experience from Disclosing**	**12.4%**	**30**	**8.5, 17.2**
Not supported by manager	66.7%	20	47.2, 82.7
Unfavorable impact on workplace relationships	26.7%	8	12.3, 45.9
Felt uncomfortable/embarrassed	30.0%	9	14.7, 49.4
Lost job as a result	46.7%	14	28.3, 65.7
Negative effects on career	23.3%	7	9.9, 42.3
Treated differently	23.3%	7	9.9, 42.3
Had no choice to tell	6.7%	2	0.8, 22.1
Chose not to tell disclose to manager^*^	**100%**	**89**	
**Positive experience from not disclosing**	**84.3%**	**75**	**75.0, 91.1**
Prefer to deal with it alone	46.2%	36	36.3, 59.8
Feel uncomfortable or embarrassed	20.0%	15	11.6, 30.8
Not treated differently	24.0%	18	14.9, 35.3
Did not have a negative effect on career	24.0%	18	14.9, 35.3
Did not affect work performance	38.7%	29	27.6, 50.6
**Negative experience from not disclosing**	**15.7%**	**14**	**8.9, 25.0**
Needed to hide	7.1%	1	0.2, 33.9
Did not get accommodations	7.1%	1	0.2, 33.9
Did not get support	50.0%	7	23.4, 77.0
Could not be a positive example	11.8%	2	1.5, 36.4

* *Categories do not sum to 100% because respondents identified all factors that applied*.

### Consequences of Not Disclosing

The majority [84.3% (*n* = 75), 95% CI: 75.0–91.1] of workers who chose not to disclose reported their non-disclosure experience was positive. The main reasons they found it a positive experience were that they preferred to deal with the issues alone [46.2% (*n* = 36), 95% CI: 36.3–59.8] and the belief that their work performance was not affected [38.7% (*n* = 29), 95% CI: 27.6–50.6]. Those who had a negative experience attributed it to not receiving support [50.0% (*n* = 7), 95% CI: 23.4–77.0].

## Discussion

Our study sought to understand the experiences of workers who have had mental health issues and their decision about whether or not to disclose their struggle to their managers and the consequences of that decision.

### Deciding to Disclose

Our results indicate that about three quarters of workers disclosed their mental health issues to their managers. These results fall within the range reported in the literature. For example, Jones' (16) review of the literature found that reported disclosure rates ranged from 35 to 87%. In addition, a US study reported about 77% of those with a disability disclosed their disability to their manager (15). Furthermore, the proportion who actually disclosed is very similar to the proportion of workers who had no experience with mental health issues but thought they would disclose if they did (24).

The reasons workers in our study sample attributed for their decision to disclose were consistent with the literature from other countries (10, 15, 22). It appears that feelings play an important role in decisions. Specifically, feelings about managers contribute to the decision to disclose. Positive feelings encouraged disclosure. In addition, feelings of responsibility to their workplaces were another significant contributor. This may also reflect a perceived alliance and shared values with managers.

#### Positive Experience With Disclosure

The majority of those who disclosed found it a positive experience. They represented the largest group in our sample. This suggests that the majority of managers were able to work effectively with employees who disclosed their mental health issues. Indeed, the majority of respondents who reported having a positive experience attributed it to the support they received from their managers. This may be a reflection of the incentives the Dutch system creates for employers to prevent work disability. Under the Dutch Gatekeeper Protocol legislation, employers are financially responsible to provide sickness absence benefits for 2 years regardless of the cause of the absence (25). As a result, Dutch employers may be proactive in disability prevention (25). Future work could focus on dyads of workers and their managers to understand the experience from the manager's perspective. What organizational contexts enable the positive interactions?

One might argue that these results suggest that stigma is not a “real” problem in workplaces. We would respectfully submit that there is still a proportion of workers who either do not disclose or who do not have positive experiences with their disclosure decisions. These workers may be affected by stigma. If this is the case, workplaces seeking social justice for all workers still have work to do to address stigma. One of the ways stigma is addressed is through workplace interventions.

#### Negative Experience With Disclosure

We also found about 12% who disclosed and did not have a positive experience. This is consistent with findings reported by von Schrader et al. (15) who reported that about 10% of their respondents who had a disability reported negative disclosure experiences.

In our study, negative disclosure experiences were attributed to extrinsic factors such as not having managerial support or losing their jobs. Experiences such as these indicate that some workers were in workplaces without support. Indeed, van Sonsbeek and Gradus (26) reported that there appeared to be a variation in the effects of the Dutch Gatekeeper Improvement Act among business sectors and the company sizes. van Sonsbeek and Gradus (26) and others suggest (27) the variation may be related to differences in the amount of resources the organizations invest in observing the legislation. Kopnima and Haafkens (28) note similar findings. They observed heterogeneity among and within Dutch organizations in how disability policies are interpreted and implemented. They point out that the flexibility of Dutch legislation allows organizations to be responsive to the individual needs of workers. At the same time, this can lead to inconsistently implemented policies. This may indicate a need for education and continual guidance within organizations to ensure the observance of national anti-discrimination legislation.

### Deciding Against Disclosure

About a quarter of our sample decided against disclosing. Intrinsic factors played a relatively more significant role in the decision than extrinsic factors. The most significant reason for non-disclosure was the preference to deal with it alone.

#### Positive Experience With Non-disclosure

The majority of workers who did not disclose attributed their positive experiences to intrinsic factors such as being able to handle the issues alone. These responses may be the result of the fact that there are workers who are able to handle issues by themselves. For example, the literature suggests that there are occupations in which workers have the autonomy to make work adjustments (17). In their study of Dutch workers, Boot et al. (8) found that less than a quarter of workers with mental disorders required work adjustments. In those circumstances, workers would not necessarily need to disclose. Nevertheless, they might benefit from education that helps them to effectively self-adjust their work when they are struggling. Future research could explore the types of occupations and job content to which this could apply.

#### Negative Experience With Not Disclosing

The smallest group in our sample were those who did not disclose and had a consequent negative experience. The primary reason reported for the negative experience was that they did not receive support. This experience could be a reflection of existing workplace stigma. These workers may not have disclosed because they anticipated that there would be no workplace support. If this were the case, anti-stigma education for both managers and workers would be important.

Also, part of building a positive environment could be addressing microaggressions. Microaggressions are indirect negative behaviors that have been shown to have significant negative impacts (14). An example would be negative general comments about people with mental disorders that are not directed at a specific person. Yanos (14) points out that while these types of comments are not discriminatory, they can be discouraging and suggest that it leads to anticipated rejection. In turn, this can result in reduced likelihood of help seeking.

Microaggressions are also closely linked to self-stigma. If colleagues make derogatory comments about mental disorders and those who experience them, workers may internalize the comments. Corrigan and Rao (20) suggest that self-stigma can be addressed by teaching the worker techniques to reduce self-stigma. If there are microaggressions in the workplace, in addition to general training, it may be important for occupational health physicians to address potential self-stigma as part of treatment.

### Strengths and Limitations

This is one of the first Dutch studies to examine the experiences of workers with mental health struggles with workplace stigma. One of strengths of the study design is that it allowed participants to remain anonymous. As result, it decreased the risk of social desirability bias.

Another strength of our study is its examination of the factors that contribute to disclosure decisions. This information helps in the development of interventions to support worker decision making. For example, if an organization's goal is to increase disclosure, extrinsic factors may not be the most effective ways of encouragement. Rather, it may be the intrinsic factors that must also be addressed. These include helping managers and workers build relationships that help satisfy a need for connection and address work satisfaction (23).

At the same time, our results should be interpreted within the limitations of our data. The fact that the sample is from the Dutch context raises the question of the generalizability of the findings to jurisdictions in which employers are not directly financially accountable for the disability of their workers such as in North America.

Our data also did not include information about respondent characteristics such as respondents' job sector or occupation or race/ethnicity. These factors may have been associated with respondent work experiences and disclosure decisions.

Another limitation is that it is not possible to determine the severity of the mental health issues with which the workers struggled. In future work, it would be important to understand how the severity of issues influences disclosure decisions and consequences. For example, there is evidence that severity of symptoms is associated with help seeking (9). This raises the question of whether this is also the case for disclosure.

The data also did not contain information about past disclosure experiences. These past experiences could have contributed to reporting decisions. Furthermore, it is not possible to determine whether respondents are reporting about experiences in a current or past workplace. In this respect, the data only reflect one experience and do not tease out why respondents identified specific factors contributing to their decisions.

Finally, respondents were not asked about either the nature or timing of the disclosure. For example, as McDonald-Wilson et al. (29) note, there are levels of disclosure that include full disclosure, selected disclosure, and targeted disclosure. There are also different timings of disclosure that include at employment and waiting until help is needed. The level and timing may affect the disclosure experience (30). For example, two small studies have found that managing and planning disclosure significantly affects work outcomes (31, 32). To further enhance educational content, future work might consider types of disclosure, their timing, and the outcomes associated with each.

## Conclusions

Our results reflect the complexity involved with developing interventions to address stigma in the workplace. They suggest that addressing stigma at work may not be as straightforward as requiring all employees to receive one type of anti-stigma education. However, they do underscore the importance of worker-manager relationships. In turn, this suggests manager training regarding stigma may be warranted.

Understanding both the factors that contribute to disclosure decisions and the consequences of decisions helps to better target workplace educational programs. One correct answer to tackle this challenge does not exist. Rather, the correct answer depends on the workplace circumstances. Future research is needed to understand the optimal ways for workers struggling with mental health issues to ask and receive help if they need it.

## Data Availability Statement

The data analyzed in this study is subject to the following licenses/restrictions: The data that support the findings of this study are available from CentERdata but restrictions apply to the availability of these data, which were used under license for the current study, and so are not publicly available. Data are however available from CentERdata upon reasonable request. Requests to access these datasets should be directed to e.p.m.brouwers@tilburguniversity.edu.

## Ethics Statement

The studies involving human participants were reviewed and approved by University of California IRB. Written informed consent for participation was not required for this study in accordance with the national legislation and the institutional requirements.

## Author Contributions

EB obtained the funding to access the data and led the design of the data collection instrument for the special module. CD led the analysis, data interpretation, and manuscript writing. JW and MJ contributed to the design of the data collection instrument and contributed to the data interpretation and manuscript writing. PG contributed to the data interpretation and manuscript writing. All authors have read and approved the submitted version.

## Conflict of Interest

The authors declare that the research was conducted in the absence of any commercial or financial relationships that could be construed as a potential conflict of interest.
